# Real or Illusory? Case Studies on the Public Perception of Environmental Health Risks in the North West of England

**DOI:** 10.3390/ijerph7031153

**Published:** 2010-03-18

**Authors:** Alex G Stewart, Paolo Luria, John Reid, Mary Lyons, Richard Jarvis

**Affiliations:** 1 Cheshire & Merseyside Health Protection Unit, Suite E, Moorgate Point, Moorgate Road, Knowsley Industrial Park, Kirby, Merseyside, L33 7XW, UK; E-Mail: richard.jarvis@liverpoolpct.nhs.uk; 2 Centre for Public Health, Research Directorate, Faculty of Health and Applied Social Sciences, Liverpool John Moores University, Kingsway House, Hatton Garden, Liverpool, L3 2AJ, UK; E-Mails: p.luria@ljmu.ac.uk (P.L.); john.reid@hpa.org.uk (J.R.); m.lyons@ljmu.ac.uk (M.L.)

**Keywords:** risk perception, environmental hazards, case studies, public health

## Abstract

Applied research in a public health setting seeks to provide professionals with insights and knowledge into complex environmental issues to guide actions that reduce inequalities and improve health. We describe ten environmental case studies that explore the public perception of health risk. We employed logical analysis of components of each case study and comparative information to generate new evidence. The findings highlight how concerns about environmental issues measurably affect people’s wellbeing and led to the development of new understanding about the benefits of taking an earlier and more inclusive approach to risk communication that can now be tested further.

## 1. Introduction

Throughout the North West of England there is a strong heritage and legacy of old industrial sites that, together with industrial developments and social issues emerging in more recent years, has led to the recognition of significant environmental hazards to health.

The North West of England was affected early by the developments of the Industrial Revolution. In the space of 150 years, between 1750 and 1900, the life of England was transformed and in turn, this revolution changed the whole of the world. The mechanisation of the textile industries in the cotton mills of Lancashire, advances in iron-making techniques and the increased use of underground coal, came together with the introduction of canals, improved roads and railways to make industrialisation and its resulting trade possible. Even inland Manchester was linked by waterways to the sea, some 30 miles away, and competed successfully with Liverpool as a port for many years. Heavy industry, such as shipbuilding on the River Mersey, became a major employer. As industrialisation grew in extent and diversity, local industry in Cheshire expanded from soap manufacture to the mass production of chemicals (1863) and the first plant capable of producing industrial quantities of chlorine (with by-products of caustic soda and hydrogen) from brine started production in Runcorn in 1897. The modern chemical and plastics industry had been born.

The complex interactions between the environment, whether industrial, social or natural, and health has been widely highlighted and studied by many authors and are now very well acknowledged [1,2]. While the early work concentrated on occupational exposures to chemicals [3] more recent work has focused on quantitative risk assessment associated with wider environmental hazards [4].

The public perception of risk within these interactions between chemicals, the environment and health can perplex many, especially those raised who rely heavily on the medical model of health. Diefenbach & Leventhal [5] remarked that “*it is not uncommon for a person to feel ill and complain about symptoms without any physical signs of a disease. In these cases*, *the medical model is unable to provide explanations that satisfy either patient or practitioner*”.

Health risk assessment related to environmental hazards identifies, estimates, and assesses the potential impacts of factors in the environment on human health, whether directly or indirectly, and is used by professionals with responsibility for public health protection and policy makers as evidence to support their decisions [6]. For example, World Health Organization standards [7,8] arising from quantitative risk assessments have led to legislation in Europe [9] and beyond which has contributed to a reduction in atmospheric pollution.

Risk assessment has been regarded as an objective interpretation of risk, based on the principle that all risks can be expressed numerically, allowing them to be compared and prioritised, sometimes to the extent that it gives the illusion of complete control over a risk.

Risk assessment, in objective, “scientific” (numerical) terms, relates to an estimation of the likelihood of an adverse health event and of its consequences should it occur, measured in terms of economic loss, physical damage or human injury. Its calculation depends on how accurate and comprehensive the initial information available is and its applicability to the situation under investigation. A generally accepted quantitative expression of risk is the product of the magnitude *M* of an unwanted event and either its incidence or frequency *F* or probability *P* of occurrence, usually per year [10]. Frequency is the occurrence of the same event per unit time, while the magnitude represents an attempt to quantify the health consequences per event. Relative Risk is a commonly used alternate measure that indicates the relative incidence of an unwanted event occurring in one group of people compared to another. To many people, a 200% increase in risk sounds shocking, but when the initial risk is very small, doubling it may still represent a very low risk. Relative Risk is a measure that can easily confuse when used in discussions with the public.

This quantitative approach to risk assessment fails to take into account the wider health and social implications associated with any particular situation, which go beyond any statistical interpretation of unwanted events and the economic acceptability of their consequences. It is likely that such numerical approaches only ever partially assess the risk. Similarly, quantitative approaches do not always have a clear meaning for the general public, who usually consider risk as a specific circumstance of life, most often unwanted, where quality of life, if not life itself, is somehow threatened. There is a strong emotional element in this and, probably for this reason, scientists tend to label it as ‘perceived risk’. However, it is easy to fall into the trap of describing the first approach as ‘real’ and the second as ‘irrational’ and the two as irreconcilable or juxtaposed [11–13].

More logically, risk has two facets that are reflected in how it is approached as well as how it is defined and evaluated [14]. The first aspect refers to the desire of policy makers and others to predict the potential impact of some events. The second refers more to lay people’s estimation of the significance of these outputs, and their expectations in terms of quality of life. According to some other authors, this duality is also reflected in the distinction between ‘risk assessment’ and ‘risk evaluation’. This distinction remains controversial [9,10] as it can have clear social and political implications. As Fishhoff *et al*. [10] noted, “*No definition is advanced as the correct one*, *because there is no one definition that is suitable for all the problems. Rather*, *the choice of definition is a political one*, *expressing someone’s views regarding the importance of different adverse effects in a particular situation*”.

Today, public health organisations are encouraged to acknowledge the fundamental contribution that perception and communication have to risk management [15]. Public health and environmental health practitioners are expected to take a holistic approach to risk management, in order to understand the needs of the community, to communicate effectively with the community on its own terms and to successfully involve the public in any relevant risk assessment (such as an Environmental Impact Assessment or a Health Impact Assessment).

In the last few years, there has been an increasing interest in risk perception and communication in the private and public sectors, as well as in academia. Complex studies, in particular in the fields of economics, social science, engineering and psychology, investigate the delicate and composite way people perceive risk and deal with it, and these are used to inform environmental and health professional practice. From a practical point of view, it is as important to understand the needs and expectations of a population affected by a local environmental health hazard, as it is to predict the health risks in detail, since it is this population which is likely to experience most of both the direct and indirect negative effects of an environment under stress.

This study was undertaken to advance the understanding of the perception of risk in communities in the North West of England exposed to a variety of environmental situations which are possibly or inherently risky to health. It seeks to use and to justify a case series approach.

## 2. Methods

### 2.1. The Case Study Survey

In 2007−2008, the authors undertook a survey of situations recently dealt with by environmental and public health practitioners in the North West of England, focusing on the risk perceptions of environmental health hazards. The project was developed as part of the annual work plan agreed for the Health Protection Agency North West’s environmental and chemicals team.

The work aimed to provide public health practitioners and policy makers with a useful document to assist in the practical management of public concerns in relation to potential environmental hazards, identifying lessons learned, examples of best practice and areas that need further development.

The study did not intend to prove any particular hypothesis on risk perception, nor did it include any primary survey data. Rather, it explored the importance of public perception of risk through the content analysis of ten case studies. The methodology employed logic and matching of information to generate new evidence.

In particular, it:

- Identified and explored environmental health hazards using a case study approach
- Compared public perception of the level of risk posed, with best evidence available about known health risks associated with a wide range of hazards
- Collated evidence and made recommendations for appropriate communication activities
- Produced a list of resources to help professionals understand community concerns and develop strategies to manage environmental risks

The work was organised into four main phases. In the first phase, the steering group formulated the objectives, identified the possible stakeholders, and suggested ten major areas of environmental concern in the region:

- Waste facilities (differentiated into landfills, incinerators, composting facilities)
- Contaminated land
- Chemical incident
- Traffic
- Air quality
- Water quality
- Food
- Flooding
- Power generation (including supply systems)
- Radiation (differentiated into ionising and non-ionising radiations)

These areas of concern were considered to be significant, but not a comprehensive picture of the regional situation and so respondents were encouraged to suggest any other environmental hazards, which they considered noteworthy and/or frequent in the region.

It is important to note that case studies, areas of concern and related issues utilised in this work were suggested on the basis of everyday practice. In other words, the study is based on expert opinion. In this way, the work has its start and end point with health practitioners: they provided the topics that have been developed into a report intended for them.

In the second phase, the questionnaire was designed and tested. This resulted in a two-page semi-structured questionnaire composed of three main sections:

1. Specific information about the hazard, *i.e.*, type of hazard and area of concern, its setting and timescale (four items)
2. Description of the public’s reaction and the public authorities’ response (three items)
3. Any ongoing problems (three items)

Practitioners were asked to submit supporting documents and any publications relating to them in the peer reviewed or grey literature.

The questionnaire was administered to 30 environmental and public health practitioners in the North West of England. For the majority of them, it was necessary to send out reminders and, in a few cases, telephone calls were made. In total, 17 case studies were collected, between July and December 2007.

In the analytical phase, the case studies were grouped by the main areas of concern, following content analysis. This grouping was functional, in order to adequately cover each topic. Given that both hazards and risks are multi-causal, the steering group acknowledged that any attempts to classify risk perception using any other simple formula based on causality was likely to fail.

As expected, the number of cases submitted for each area varied and studies were selected for inclusion on the basis of their perceived general usefulness and other practical criteria. In particular:

- No more than two cases were presented for each topic
- Cases not supported by enough information and topics not supported by any case were excluded

Two cases were also excluded because of ongoing confidentiality needs. As a result, the number of examples fully analysed and included in the final report fell from 17 to 10, covering only seven of the 10 areas of concern initially proposed. It is difficult to establish whether the lack of cases in some areas was due to technical reasons (such as the difficulty in accessing information because of its confidentiality) or the topic being considered less significant by the practitioners.

The case studies were then analysed paying attention to the public perception of the hazard, any authorities’ response, and the outcomes. Examples of specific factors examined were:

- Type of hazard (natural or technological), and people’s familiarity with it
- Number of people involved
- The inequity of the distribution of the risk (e.g., some social groups may be more affected than others)
- The socio-economic background of the involved population (e.g., levels of deprivation)
- The presence of vulnerable groups such as small children or pregnant women
- The media coverage
- The type and timescale of authorities’ response
- Authorities’ communication and engagement with the public
- Type of information provided to the local population
- Use of statistics and toxicology to communicate risks
- People’s satisfaction with authorities’ response

### 2.2. Literature Review

Simultaneously, a critical literature review was carried out for each area of concern, to identify:

- The best evidence available regarding perception of the given hazard
- The quantitative and qualitative levels of risk posed to human health by the given hazard
- Factors which may help understand community concerns and anxieties associated with the hazard

Academic resources (Academic Search Complete, Medline and ScienceDirect—information sources for scientific, technical, and medical research) were searched; a free web search using Google and a search of official guidelines, by authors and key words were also conducted. The most important key words used were “risk perception” and “public perception” in conjunction with the ten major areas of concern in the region.

More than 300 documents from peer reviewed and grey literature were collected and reviewed, identifying 88 relevant documents which were then classified into the previously identified major areas of concern. The information gathered was then compared with the content analysis of the case studies, to highlight discrepancies between the public and expert understanding of each hazard and to identify key issues that could be used to develop strategies to manage environmental risks more effectively.

A final report was produced for wider distribution among environmental and public health practitioners. This included:

- An overview of the concept of risk perception
- An account of each case study, mirroring the questionnaire’s structure, *i.e.*, type of hazard, setting and timescale, public’s reaction, authorities’ response, and any outcomes
- A commentary on each case highlighting the most important concepts that may be helpful in understanding the health and social problems arising from the public perception of these particular hazards
- Recommendations for practitioners, focusing on lessons learned
- A complete list of references organised both by case study, author and areas of concern, to facilitate the search for useful resources on each specific area of concern

The report was designed primarily to support organisations in the North West of England, who deal with local groups and communities on a daily basis. However, we recognise that the findings from the case studies may have national or international relevance, hence the dissemination of the work and findings in this paper.

## 3. Results

Ten studies met the criteria for inclusion (Table 1). They covered waste incineration, land contamination, odour and air contamination, non-ionising radiation, acute chemical incidents, flooding and cancer due to environmental risks.

There were no cases covering traffic, water contamination or power generation, although power generation was part of the planning application for the waste incineration case (Ince Resource Recovery Park). The issues of public concern around Ince focussed more on waste incineration than on power generation, hence the classification used here.

Several cases revolved around new developments, located in areas with medium to high levels of deprivation. Most of the concerns identified across all the case studies related to land or air contamination, often focusing on cancer as the most feared output. Issues about uncertain outcomes were of most concern wherever children were involved.

The response of the various authorities dealing with the hazards was often limited to the quantification of environmental or health risks and involved statistical analyses. In about 50% of the cases there was public dissatisfaction with the authorities’ reaction, revolving around undue delay, or a response that was too technical and did not take into account intangible factors, such as emotional issues, that are important to the general public.

Overall, there was a lot of similarity in the issues arising from the cases (Table 2) and those reported in the literature (Table 3). The areas where this study adds to the literature include the highlighting of differing perceptions and needs of the professionals and the public and the resulting anger and distrust. As a consequence, it is important to assess community anxiety and stress as a means to assessing relationship issues, resulting communications and the role the community can and should play in responding to environmental hazards to health.

Relationships between the community and the authorities were perhaps the main key to understanding and responding to public concern. Where good relationships allowed involvement of the community by the authorities and where good, clear communication with the community took place (e.g., acute myeloid leukaemia in Leftwich where the authorities listened to the community concerns and addressed community needs as well as their own with direct participation of the community in the project) the results of investigations were more likely to be accepted (even if not liked) than where the issue had a long history of poor relationships or fixed ideas (e.g., Malkins Bank golf course).

Similarly, where the investigation by the authorities produced a clear positive finding which accorded with the view of the community (e.g., Sandon Dock) there was more likely to be greater satisfaction and acceptance of the authorities’ response than where negative findings had to be conveyed (e.g., West Bank cancer cluster) or where the focus of the authorities was at odds with that of the community (e.g., Flooding in Carlisle).

## 3. Discussion

The investigation of risk perception based on ten diverse case studies in the North West of England highlights the differences between the authorities dealing with potential environmental hazards to health and the communities they serve. Despite the diversity of the cases studied and the wide range of hazards involved, there are clear and consistent findings relating to distrust of professional and regulatory bodies, inadequate or inappropriate communication, lack of recognition of health effects relating to risk perception and response, and an inability of professionals to adequately understand and tackle public anxiety and anger.

A case study approach has a lot to offer in developing understanding of complex environmental problems and the perceptions of risks that drive responses by both communities and authorities, particularly since a variety of cases were included. Nevertheless, this approach would benefit from a more rigorous theoretical framework [48] since theoretical and methodological collaboration between the more numerical sciences such as epidemiology and ‘softer’ social sciences is important for public health, modifying both fields in a positive and helpful way [49].

Much modern scientific investigation and professional practice in health, including those involving environmental issues, still sees explicit methods, numerical analysis or standard operating procedures as the gold standard. Many years ago, Polyani [50] identified tacit knowledge as playing an important role in scientific investigation. This raises questions about a simplistic application of the hierarchy of evidence to Public Health [51] and risk perception issues while at the same time strengthening the case for the use of several, disparate case studies as a series to enhance the evidence base in risk perception.

Tacit knowledge and skilled judgement are essential in any aspect of life, including scientific and professional practice; they allow the practitioner to take account of particulars that may affect the situation under review. No real world investigation without a control group can allow or account for every confounder or determinant; no response can take every viewpoint and perception into account. But to underestimate perceptions differing from one’s own [52] is as bad practice as to overlook confounding in epidemiology.

Many of the case studies involved examination of public reaction to new developments and it is possibly in this area in particular that the lessons learned from this analysis will have greatest impact. Failure to understand and tackle public perception and concerns about risk at an early stage increases the chance of negative public reaction, and may be used as a justification for attempts to block the development. Lessons from the literature about the different perspectives are slow to be translated into daily practice. Bennett and Calman [15, p. 3] wrote in 1999 that *“there has been a progressive change in the literature on risk from an emphasis on ‘public misperceptions’ …to approaches which stress that public reactions to risk often have a rationality of their own*, *and that ‘expert’ and ‘lay’ perspectives should inform each other as part of a two-way process”.* Heated disagreements between public authorities and the local community ensue and whichever side “wins”, such struggles inevitably lead to distrust on both sides, considerable delay, unnecessary expense and problems gaining planning and other permissions not only for this development, but also in the future.

By concentrating solely on risks to physical health, professionals from both public health and regulatory bodies fail to understand and take into account the wider determinants of public health and wellbeing. The combined case study approach facilitates the synthesis of lessons learned and the discovery of consistent patterns and leads to recommendations about improving risk communication and situational management that may enhance the professional and public perception of risk and promote health. The approach may also stimulate more traditional epidemiological thinking by suggesting new areas of research or approaches. Not least, it enhances the desirability of developing the Bayesian approach to quantitative risk assessment of environmental risks to health.

Case control studies, randomised controlled trials and other statistical and narrowly focussed approaches provide a mathematical assessment of the probability of an untoward event occurring depending on various exposures. They certainly have their place but even the best are not, and cannot be, the whole story since they cannot take the complexities of the human situation into account.

Although this work is based on a desk-top review without engagement with the general public, which may be regarded as a weakness, the cases selected were ones which had enough information in them to enable identification of the major issues. The process also facilitated assessment of the interaction between the community and the various professional officers of the bodies who dealt with them.

This work highlights the cross-cutting nature of many of the issues relating to risk perception of environmental hazards as well as the slowness of discussions in the literature moving into daily practice. For example, distrust of authorities was commonly reported even after detailed investigations had been carried out. Distrust may be an indicator of a lack of common understanding. Debates by professionals about whether public concerns were justified, or whether any hazard actually existed, can indicate a lack of understanding of the effect of anxiety on the public wellbeing. This in turn can undermine the professional’s ability to listen and respond sensitively to public concern. Professionals need to trust the public if they wish the public to trust them [15].

In several case studies, a direct result of the authorities’ rather negative approach to the public perception of the risk was a breakdown in communications and the relationship between the public and various health and local authority professionals involved. Communication breakdown is unfortunately a common feature of situations with a long history of poor interaction between the authorities and the community and such situations are difficult to overturn. In the case of Malkins Bank golf course, a number of years of disagreement between parts of the community and the local authority meant that four different attempts were made to examine the claims of a cancer cluster, but each one was greeted with disbelief that was coloured by the previous poor relationship.

Wherever the community was involved in one way or another from the beginning, and their views listened to and considered, as in the investigation of the acute myeloid leukaemia cluster in Leftwich, then even negative findings were more easily accepted. Swift and relevant communication by a variety of means and the offer of help in cleaning up asbestos debris after the serious fire at Greenall’s distillery resulted in only one dissatisfied resident contacting the local authority.

Risk perceptions are not changed simply by improved communication from professionals to the public. Risk communication is not simply a one-way flow from sources of information about the risks posed by environmental hazards to health (scientists, agencies, interest groups, eyewitnesses) through transmitters who amplify the message (media, institutions, interest groups, opinion leaders) to receivers who accept the information (general public, affected people, group members, those exposed), but a two-way exchange, or even dialogue between all parties [15, pp. 66–69]. Problems and solutions are not found only in one group or another, but in all.

“Mental models” have been suggested as a means to integrate community and professional perspectives and knowledge into an effective communication strategy [53]. Following a literature review and interviews with experts, as in this study, semistructured interviews with members of the public are used to develop two conceptual models, one expert and one lay. These are then compared to identify important discrepancies which are then measured with a structured survey instrument that provides a rigorous baseline measure of the gaps in public understanding. Finally, a communication protocol is developed to address the knowledge gaps that influence important decisions by the public [54,55]. Such an approach should not stop with the development of a communication strategy but should be extended to incorporate full community involvement and participation in addressing the issues around risk perception and risk management of environmental hazards to health.

## 4. Conclusions

The analysis of the findings from several case studies facilitated the integration and synthesis of information from disparate situations in a way that statistical analysis cannot. This technique enabled very clear lessons to emerge about how risk perception of environmental hazards causes anxiety which has a significant impact on public health and that professional debates about the statistics are of little interest, nor use to assist the public understanding of environmental hazards. The dismissal of public concerns because they are not supported by statistical evidence appears to generate distrust rather than offer reassurance. Undoubtedly, one of the key conclusions from analysis of the case studies is that good communication and public involvement from an early stage is essential for generating trust and that when this happens, even though the outcome of an investigation is not what is expected, or hoped for, it is accepted by the public.

The use of disparate case studies, in a linked series, generated recommendations about how public health could be improved by understanding the public perception of risk. Further work of a similar nature would serve to improve understanding, augment the applicability of recommendations made, and strengthen responses to and management of environmental hazards to health.

## Figures and Tables

**Table 1.** Details of the cases used in the analysis of perception.

**Area of concern**	**Case study**	**Short description**	**Public risk perception**	**Authoritie’s response**	**Outcomes**	**Public satisfaction**	**Additional notes**
Incineration of waste	*Ince Resource Recovery Park Ince*, *Cheshire*	In 2006, a private company submitted a planning application for a waste management park, which included a Refuse Derived Fuel (RDF) power plant.	General health concerns with some distrust of the siting of the RDF plant and concern about wider social issues.	The Primary Care Trusts (PCTs) commissioned a rapid Health Impact Assessment (HIA) exploring these concerns.	The HIA concluded that major effects on health were not expected from the incinerator itself, but from its planning application, since it raised high levels of anxiety and stress in the local population.	Residents were not completely satisfied because some of the HIA’s recommendations were not taken into consideration by the planning authority.	The application was initially rejected by the planning authority for technical reasons. A revised proposal was re-submitted and approved.
Land contamination	*Malkins Bank Golf Course Congleton*, *Cheshire*	A former industrial site was reclaimed and turned into a golf course in the 1980s. In the 1990s, the drainage system was found to be chemically contaminated. In 2002, an environmental investigation was started by the Environment Agency (EA) and local authority.	Nearby residents’ concerns about a possible cancer cluster.	The PCT, the Merseyside & Cheshire Cancer Registry carried out statistical analyses of cancer rates in this rural community. The local authority re-instituted a liaison committee which had fallen into abeyance. It included the EA, Health Protection Agency (HPA) as well as a variety of community and public representatives.	The statistical analyses concluded that there was no excess of cancer cases or of specific cancers. The environmental investigation concluded that there was no evidence of any significant risk to human health.	Residents were not fully satisfied and some still believe that there is a real health problem. The analyses were criticised by the public because they focused solely on statistics and were perceived to have covered too wide a geographical area.	Recently, the residents asked for a further review of cancer in the area. This has been carried out by the HPA and concluded that there is no evidence of a cancer cluster.
Land contamination	*Housing Development at Thingwall Hall Knowsley*, *Merseyside*	In 2002, an application was submitted for land reclamation and residential development of an old waste tip.	General concerns relating to increase in traffic and anxiety about the underground movement of old, hazardous waste in the ground	The initial application was rejected as traffic issues were not well addressed. The planning committee also required a Pollution Prevention and Control permit for waste re-deposition.	Despite the committee’s reservations, planning permission was granted on appeal in 2007 and remediation works are expected to start as soon as possible in order to meet the statutory deadlines.	Residents remain concerned about the potential for traffic problems, exposure to toxic dusts and groundwater contamination	There is a long history of redevelopment applications, submitted since the 1980s, which have been refused or withdrawn.
Odour and air contamination	*Sandon Dock wastewater plant Liverpool*	In 2000, a new biological treatment stage was introduced in the plant. This increased the amount of unpleasant odours and new measures for odour abatement were also installed.	Complaints about odour, from nearby residents. 188 letters were received in July 2001. A large number of people visited their general practitioner because of potentially related symptoms.	The Director of Public Health decided to undertake a risk assessment. A multi disciplinary, multi-agency health advisory group was established to investigate the case and produce a report.	The investigation concluded that some chemicals generated by the plant were the possible cause of the odours, but their concentration levels were not consistent with the symptoms. The public’s response was probably driven by stress and anxiety.	The residents were satisfied as the operator of the plant identified a possible source of odour as a failure of the new treatment stage and new abatement measures were put in place.	The investigation did not exclude other nearby sources of odour in addition to the one from Sandon Dock
Odour and air contamination	*Clariant Work Site Cadishead*, *Salford*	In 2005, a private company undertook ground bio-remediation of a former tar works site, a process which can produce unpleasant odours.	From 2006, the nearby residents started complaining about fumes and pungent petrol-like odours, and some of them reported health symptoms.	The local authority, the HPA and the EA investigated the source of the odours and possible health effects.	The bio-remediation process was identified as the main source of the odours; however, emissions were too low to cause health effects. More efficient odour control and monitoring measures were adopted and an information campaign carried out in the area	Most of the objections quickly ceased. However, a small number of residents continued to express health concerns and report effects to their general practitioners	Most of the latest complaints came from residents who were not included in the information campaign.
Non-ionising radiations	*Local Area Petition Southport*, *Merseyside*	In 2005, a petition from a group of residents requested the council to investigate the health risks associated with living in proximity to a telephone mast.	The residents raised concerns over their quality of life and health, supported by several self-reported complaints of non-specific symptoms.	The Council set up a multi-agency working group to review the potential risks throughout the entire borough and produce a report.	The reports indicated that there were no increased health risks for residents. However, it highlighted some gaps in the knowledge and recommended adopting a precautionary approach	Despite general satisfaction, the residents reported high levels of concern and distrust in regulatory bodies	There is no shared definition of what the precautionary principle means between authorities and communities. This can lead to failure to meet community expectations, and further dissatisfaction
Chemical incident	*Greenall’s Fire Warrington*, *Cheshire*	In 2005, a fire at a distillery involved some buildings with fallout of denatured asbestos cement from the roof covering up to 1 km. from the site.	Residents within the fallout plume expressed concerns about the asbestos fallout and the deposits on their homes and gardens.	The council put in place an information campaign on asbestos to reassure and advise the residents. HPA specialists provided support to the systematic cleanup.	Specialist contractors carried out a systematic cleanup of the area on behalf of the distillery. 425 properties were also offered a clean-up facility.	Thirty residents asked the council for further information. One resident expressed a general lack of trust in the regulatory bodies because of the absence of a proper asbestos emergency procedure.	Recently, the HPA NW led the development and production of a toolkit, to guide the Public Health response in any future large scale fire involving asbestos.
Flooding	*Flooding in Carlisle Cumbria*	In January 2005, the city of Carlisle was flooded with high water levels. About 3,500 households and numerous businesses were affected and three people died.	Residents had low expectations of the risk of flooding and were not prepared. People reported high levels of anxiety and stress or even panic.	In the early stages of the event there was a large multi-agency response to address the immediate risk to life. Many reception centres were activated.	The Primary Care services were inundated with people experiencing severe psychological trauma in the post-flooding phase.	Many people were not satisfied as the response concentrated on practical and immediate issues, but the high levels of anxiety and stress in the post-flooding phase were underestimated.	This is the only case study involving a natural hazard, indicating that issues of stress and perception are not confined to man-made situations, although natural hazards are seen as less risky by the community.
Cancer due to environmental factors	*Acute Myeloid Leukaemia (AML) in Leftwich Vale Royal*, *Cheshire*	Between 2004 and 2005, two toddlers, living in adjacent homes subsequently found to be built on an old landfill, died of a very rare form of leukaemia.	Residents expressed strong concerns about the safety of the community, land contamination and the potential risk of cancer, in particular for children.	A multi-agency investigation was set up led by health professionals but with community involvement to review the whole situation. Epidemiological investigations, gas emission tests, building inspections, soil sampling and analyses were conducted.	The investigations did not uncover any other health problems. High levels of methane and problems with the gas–tight membranes under every house were found. Four families were relocated for compassionate reasons. As expected, an environmental cause of the cancer was not identified.	Most of the residents were satisfied by the authorities’ response. However, a few persons are still convinced that an environmental cause may exist.	The public was promptly and actively involved in directing and interpreting all the investigations, both epidemiological and environmental.
Cancer due to environmental factors	*West Bank Cancer Cluster Halton*, *Cheshire*	In 2006, some residents of two adjacent streets expressed concerns about a potential cancer cluster.	The residents expressed concerns about several different cancers. Potential causes were attributed to common forms of environmental contamination in the area (e.g., land contamination).	The HPA, on behalf of the PCT, undertook statistical analyses of cancer rates in the area.	The analyses did not reveal an excess of cancers of any particular type.	The residents were not completely satisfied as the borough experiences very high levels of deprivation and mortality rates.	The HPA suggested that further and better communication with the public was clearly required

Glossary: EA = Environment Agency; HIA = Health Impact Assessment; HPA = Health Protection Agency; PCT = Primary Care Trust; RDF = Refuse derived fuel.

**Table 2.** Key issues concerning perception of risk identified through the case study analysis.

Professionals from regulatory and advisory organisations bodies and agencies often debate whether public concerns are justified, and whether any physical health hazard actually exists. However, public concerns may themselves produce significant effects on the mental, physical, social and emotional wellbeing of a population but are rarely considered to be issues that should be tackled by professionals. Regulatory bodies maybe statutorily required to focus on calculated risk; nevertheless, public perception and concerns may, at times, be more important for determining priorities for health promotion and intervention. A ‘precautionary approach’ gives regulatory bodies confidence, but may highlight knowledge gaps and trigger new concerns (*i.e.*, the public may overreact to precautionary measures justified by uncertain but negligible risks). Public reaction to an environmental hazard relates more to the feared consequences of exposure, rather than the likelihood of exposure. Unfamiliar or incomplete information may lead people to form their own inaccurate though internally consistent mental picture of the situation. Risks associated with new technology are usually considered less acceptable than natural risks, such as flooding. The health and social effects of anxiety and stress arising from awareness of a potential environmental hazard are substantial in themselves, but are not systematically reported nor easily measured. Inadequate communication about a new proposal or environmental hazard can invoke anger in the community. In general, the use of statistics is not the best way to communicate about risk with members of the public since they may not appear to the public to take into account important qualitative factors around risk. Estimation of community anxiety and stress should be included as part of every risk or impact assessment of proposed plans that involve a potential environmental hazard. This is true even when the physical health risks may be negligible. Regulatory bodies are not always trusted by the public.

**Table 3.** Key relevant points from the literature review.

**Area of concern**	**Case studies**	**Key points from literature**
Incineration of waste	*Ince Resource Recovery Park Ince*, *Cheshire*	“Not In My Backyard” (NIMBYism) [16] known with incinerators [17]. Visible chimneys stigmatise whole complex [17,18]; emotions run high with possible toxins [19], children [17], associated traffic, extent beyond immediate proximity of the site [20], synergism with local industry. Confounding issues are deprivation and other local industry [21].
Land contamination	*Malkin’s Bank Golf Course*, *Congleton*, *Cheshire*; *Housing Development at Thingwall Hall Knowsley*, *Merseyside*	A few reports of demonstrable biological signs of chronic stress [22], psychosocial stress, depression and anxiety, stigmatisation, anticipatory fears for children’s future [23], relationship stresses, often generated by chronic uncertainty or lack of control [24]. Deprived populations less likely to perceive or complain about risk [25].
Odour and air contamination	*Sandon Dock wastewater plant*, *Liverpool;* *Clariant Work Site Cadishead*, *Salford*	Odour appears to amplify fears [26], may lower irritation and reporting thresholds [27] and provokes sensory responses and complaints [28]. Personal factors (age, sex, previous experience) affect concern [29]. Psycho-physical wellbeing can be adversely affected without clear link of odour to health hazard [30]. Stress may give rise to physical problems (e.g., muscular tension, irritability, somatic anxiety) [31].
Non-ionising radiations	*Local Area Petition Southport*, *Merseyside*	Distrust of UK sources of information on radiation risk [32], with NIMBYism common [33]. Perception related to personal (e.g., age, education, gender, familiarity with technology) & external factors (e.g., lack of control, imposition of telecommunication mast/station, dread of bad effects) [34]. Precautionary measures may trigger concern [35]. Non-specific symptoms attributed to electromagnetic fields by 1–2% of population [36].
Chemical incident	*Greenall’s Fire Warrington*, *Cheshire*	Chemicals [37] misunderstood more than physical hazards, with the media playing important role [38]; certain substances highly emotive [19]; warnings and precautions can amplify concerns [39]. Dread outcomes worse than unknown, fear of catastrophe or long lasting effect [40]. Concerns over cumulative effects of small quantities raise anxiety [17].
Flooding	*Flooding in Carlisle Cumbria*	Fewer papers on perception of risks from natural hazards than technological ones; natural hazards seen as rare, but risks frequently underestimated [10]. Unexpected events have complex, long-lasting impacts: 15–20% affected by natural disaster develop symptoms of post-traumatic stress disorder [41]. Long recovery time generates anxiety [42]. Deprived populations more likely to experience/be affected by natural hazard [21] but unclear how psychosocial effects influence perception of disease [43].
Cancer due to environmental factors	*AML in Leftwich Vale Royal*, *Cheshire;* *West Bank Cancer Cluster Halton*, *Cheshire*	Public concern about cancer appears high but little literature exists on perception of individual cancers or general fear of cancer. Cancer related anxiety is unique and supported by general beliefs that cancer is an unavoidable, single disease, causing a terrible death [44] and arising from man-made pollution, chemicals or radiation [45]. Uncertainty makes this worse [46] while the difficulties of investigation determine public discontent and distrust in regulatory bodies.

NOTES: Newest references quoted; further references are given in [47].

NIMBY = Not In My Back Yard.
